# Case Report: Late-onset BK polyomavirus-associated nephropathy with encephalitis, antibody-mediated rejection, and atypical hemolytic uremic syndrome: a rare overlap causing allograft dysfunction, therapeutic challenges, and multidisciplinary management

**DOI:** 10.3389/fmed.2026.1981467

**Published:** 2026-10-09

**Authors:** Jie Zhou, Lichuan Yang, Jiaojiao Zhou

**Affiliations:** 1 Department of Outpatient, West China Hospital, Sichuan University, Chengdu, China; 2 Department of Nephrology, West China Hospital, Sichuan University, Chengdu, China; 3 Department of Medical Ultrasound, West China Hospital, Sichuan University, Chengdu, China

**Keywords:** allograft dysfunction, atypical hemolytic uremic syndrome (aHUS), BK polyomavirus-associated encephalitis, BK polyomavirus-associated nephropathy, thrombotic microangiopathy

## Abstract

**Background:**

Kidney transplantation is the definitive treatment for end-stage renal disease. Late allograft dysfunction has complex and heterogeneous etiologies, and concurrent overlapping pathologies pose substantial clinical challenges that require integrated multidisciplinary management.

**Case presentation:**

A 23-year-old female with previously stable allograft function on maintenance immunosuppression presented with progressive clinical deterioration 6 years after deceased-donor kidney transplantation. Initial respiratory symptoms progressed to tonic-clonic seizure, neuropsychiatric disturbances and acute renal function decline. She was diagnosed with active BK polyomavirus viremia with encephalitis, complement-mediated atypical hemolytic uremic syndrome (aHUS), and biopsy-proven BK polyomavirus-associated nephropathy (BKPyVAN) plus concurrent C4d-positive antibody-mediated rejection (AMR). A multidisciplinary team delivered an individualized regimen to resolve the therapeutic conflict: 5 plasma exchange sessions, conversion from mycophenolate mofetil to mizoribine to balance viral control and rejection prophylaxis, plus adjunctive symptomatic therapies. Neuropsychiatric symptoms resolved gradually, and hematological parameters and renal function stabilized by day 29 of admission, leading to a stable discharge. However, the patient progressed to allograft failure and required maintenance dialysis at 1-year follow-up.

**Conclusions:**

This case highlights the complexity of late allograft complications with overlapping pathologies. Timely multidisciplinary consultation and balanced individualized strategies are critical to optimize clinical outcomes in such rare cases.

## Introduction

Kidney transplantation is the definitive treatment for end-stage renal disease, yet long-term allograft survival remains persistently challenged by a spectrum of late complications, most notably opportunistic infections, allograft rejection, and thrombotic microangiopathy (TMA) (1, 2). The coexistence of multiple pathologies with opposing therapeutic principles poses substantial clinical dilemmas, and optimal management typically requires integrated multidisciplinary collaboration (3–5). BK polyomavirus (BKPyV) is one of the most prevalent viral pathogens in kidney transplant recipients (6). BKPyV-associated nephropathy (BKPyVAN) classically develops within 1–2 years post-transplant, corresponding to the peak intensity of maintenance immunosuppression. However, late-onset BKPyVAN, defined as disease onset beyond 5 years after transplantation, is being increasingly recognized in clinical practice, and is characterized by more advanced interstitial fibrosis and a higher risk of irreversible graft loss compared with early-onset cases (7). While renal parenchymal involvement is the hallmark of pathogenic BKPyV reactivation, disseminated infection with central nervous system involvement manifesting as encephalitis is extremely rare in kidney transplant populations (8, 9).

Thrombotic microangiopathy is another severe and clinically challenging complication after kidney transplantation, with a reported incidence ranging from 0.8% to 15%, and it exerts a significant negative impact on both graft and patient survival (10). The syndrome is defined by the triad of microangiopathic hemolytic anemia, thrombocytopenia, and end-organ damage secondary to endothelial injury and microvascular thrombosis. Its clinical presentation is highly heterogeneous, varying from renal-limited disease diagnosed only on allograft biopsy to systemic manifestations involving the nervous, gastrointestinal, and cardiovascular systems (10). Complement-mediated atypical hemolytic uremic syndrome (aHUS) is a prototypical severe subtype of TMA driven by dysregulated overactivation of the alternative complement pathway (11). *De novo* aHUS after kidney transplantation is rare but associated with poor graft outcomes (1, 12), and intercurrent infections are well-documented triggers for disease onset. Antibody-mediated rejection (AMR) represents another major cause of transplant-related TMA (13), and the potential coexistence of these two entities further complicates differential diagnosis and clinical decision-making.

To date, the simultaneous occurrence of late-onset BKPyVAN with encephalitis, AMR, and complement-mediated aHUS in a single kidney transplant recipient has been rarely documented. Clinical management of such overlapping conditions is inherently paradoxical: reduction of immunosuppression is the cornerstone of BKPyV infection control, whereas AMR and aHUS require intensified immunomodulatory and complement-targeted therapy. Herein, we report a rare case of a 23-year-old female who developed disseminated BKPyV infection (BKPyVAN with viral encephalitis), concurrent C4d-positive AMR, and complement-mediated aHUS at 6 years after kidney transplantation. We detail the diagnostic workup, multidisciplinary therapeutic strategy, and clinical course of this case, to provide practical insights for the management of similar complex overlapping clinical scenarios.

## Case presentation

A 23-year-old female, 6 years following deceased-donor kidney transplantation for end-stage renal disease, had been on regular maintenance immunosuppression with tacrolimus 1.5 mg twice daily, mycophenolate mofetil 360 mg twice daily, and prednisone 8 mg once daily. She presented with a 2-week history of productive cough, agitation, and behavioral disturbance. To provide a clear overview of disease progression and clinical management, the timeline of clinical manifestations, diagnostic findings, and therapeutic interventions is summarized in Figure 1, and the detailed clinical course is described as follows. Two weeks before admission, she presented with pharyngeal discomfort and productive cough, which showed no improvement after levofloxacin therapy. One week prior to admission, she developed a tonic-clonic seizure followed by progressive agitation and abnormal behavior. Workup at a local hospital revealed severe anemia and acute allograft dysfunction. Neurological worsening despite symptomatic therapy prompted transfer to our center, with clinical suspicion of intracranial infection or uremic encephalopathy on admission (Day 0). Vital signs included temperature 36.8 °C, heart rate 91 beats/min, respiratory rate 19 breaths/min, and blood pressure 141/85 mmHg. The patient was delirious and presented with pale, anemic facies. Initial workup demonstrated severe anemia, thrombocytopenia, schistocytes on peripheral blood smear, and profound CD4⁺ T-cell lymphopenia. Lumbar puncture showed elevated intracranial pressure and increased cerebrospinal fluid protein without pleocytosis. Active BK polyomavirus viremia was confirmed on Day 5. Empirical antimicrobial, antiviral, intracranial pressure-lowering and antiepileptic therapies were initiated, with cautious adjustment of immunosuppression. On Day 10, the patient was transferred to the neurology department due to worsening neuropsychiatric symptoms, with a clinical diagnosis of BK virus encephalitis. She received continued ganciclovir for antiviral coverage, nifedipine for blood pressure control, levetiracetam for seizure prophylaxis, olanzapine for management of behavioral and psychiatric disturbances, and mannitol for intracranial pressure reduction. During this period, the patient exhibited intermittent agitation, involuntary smiling, soliloquy, involuntary limb movements, and aggressive behavior. Subsequent complement profiling revealed elevated soluble C5b-9 and positive anti-complement factor H (anti-CFH) antibody with normal ADAMTS13 activity, consistent with aHUS. Following multidisciplinary consultation, the patient was transferred back to the nephrology department on Day 18 for definitive management, and plasma exchange was initiated promptly. Given a subtherapeutic tacrolimus trough level, the immunosuppressive regimen was optimized, with tacrolimus increased to 1.5 mg twice daily and combined with mycophenolate mofetil 360 mg twice daily and prednisone 8 mg once daily. On Day 21, ultrasound-guided renal allograft biopsy was performed. Pathological findings indicated BK virus infection and antibody-mediated rejection. The biopsy also revealed severe chronic allograft injury, suggesting a poor prognosis and creating a therapeutic dilemma between antiviral and antirejection strategies. On Day 24, in light of the biopsy findings, mycophenolate mofetil was switched to mizoribine at a dose of 100 mg orally every morning and 50 mg orally every evening for rejection prophylaxis to mitigate the risk of ongoing viral replication. By Day 33, the patient exhibited no residual speech or behavioral abnormalities, with a decline in serum creatinine, and was discharged on maintenance immunosuppressive therapy.

**Figure 1:**
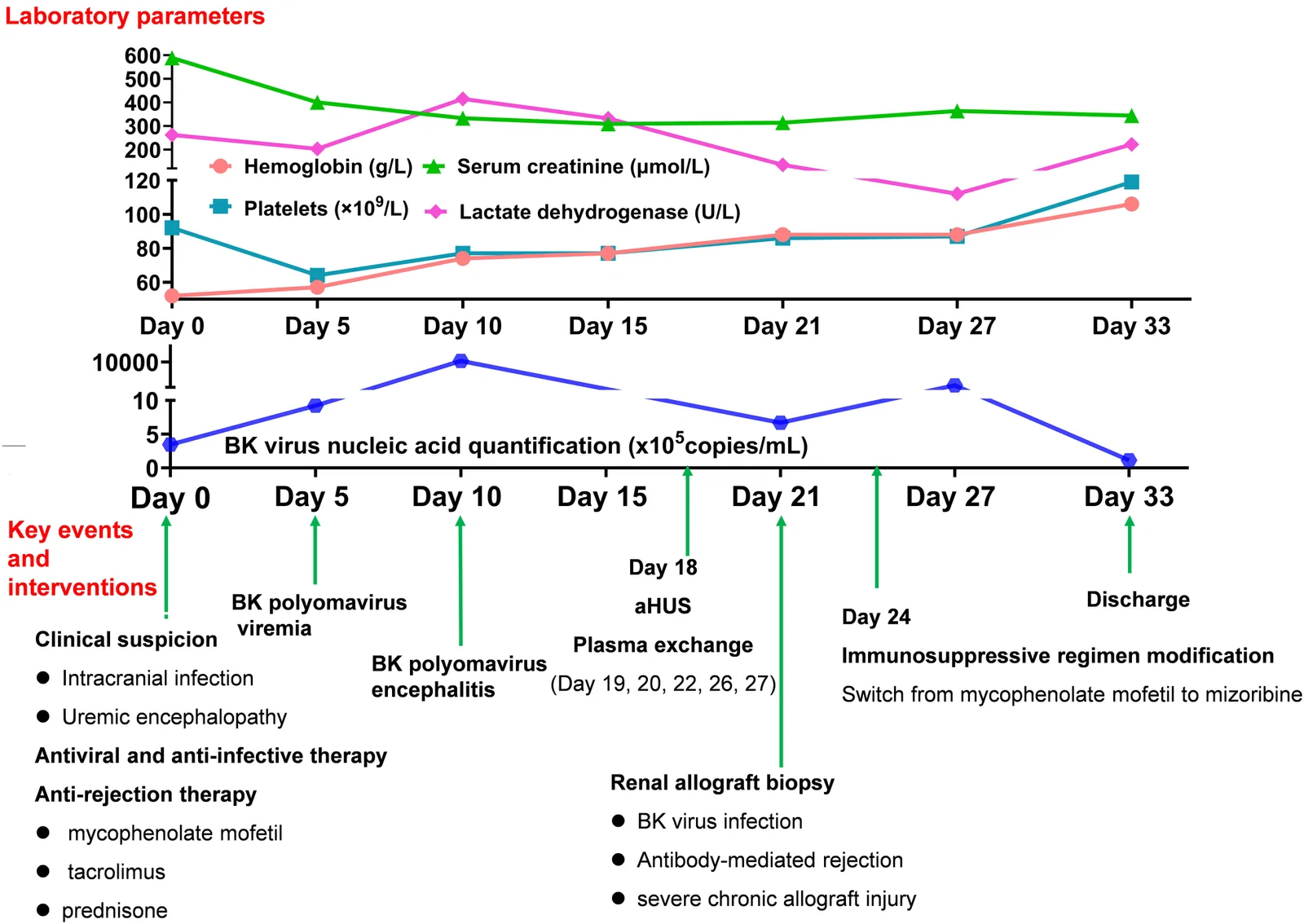
Timeline of clinical events, diagnostic findings and therapeutic interventions in the patient.

Key laboratory parameters are summarized in Table 1. Electroencephalogram showed increased slow-wave activity across all bilateral leads, interpreted as mild diffuse abnormality. Brain MRI revealed mild dural thickening and slightly widened extracerebral spaces (Figure 2). Renal allograft ultrasound identified allograft cysts, urinary salt crystals, renal calculi, and hydronephrosis. Renal allograft biopsy histopathology indicates BK virus infection as the final diagnosis, with antibody-mediated rejection not excluded (Figure 3). Immunofluorescence staining shows deposition of IgA, IgG, IgM, C3, C4, C1q, *κ* and *λ* only in 5–6 sclerosed glomeruli. Paraffin immunofluorescence staining yields negative results for IgA, IgG, IgM, C3, C4, C1q, *κ* and *λ*, while *κ* and *λ* are positive in inflammatory cells. Immunohistochemistry staining shows focal positive C4d (c4d1), positive SV40, predominant CD4-positive T cells, minority CD8-positive T cells, few CD20-positive cells, majority CD3-positive cells, CD138-positive plasma cells, negative CMV, negative IgG4, and a Ki67 proliferation index of approximately 5% to 10%. Electron microscopy revealed only one globally sclerosed glomerulus, accompanied by thickening of Bowman's capsule and pericapsular fibrosis. The renal tubules and interstitium presented moderate to severe lesions, manifesting as moderate to severe tubular atrophy, thickening of the tubular basement membrane with multifocal epithelial necrosis, and moderate to severe interstitial fibrosis with inflammatory cell infiltration, along with concurrent vascular wall destruction. Overall, the findings indicate severe tubulointerstitial injury with glomerulosclerosis.

**Table 1:** Key laboratory parameters.

Laboratory Parameters	Admission	Discharge
Hematological tests		
Absolute reticulocyte count	0.0100 × 10^12^/L	0.1321 × 10^12^/L
Hemoglobin	52 g/L	84 g/L
Platelet	92 × 10^9^/L	119 × 10^9^/L
Peripheral blood smear morphology		
Red blood cell anisocytosis	21.3%	16.1%
Schistocyte percentage	0.8%	0.1%
Direct antiglobulin test	Negative	
Complement C3	0.6630 g/L	
Haptoglobin	0.126 g/L	0.397 g/L
Serum creatinine	589 μmol/L	344 μmol/L
Lactate dehydrogenase	262 U/L	222 U/L
Tacrolimus trough blood concentrations	7.30 ng/mL	5.05 ng/mL
CMV-DNA	1.35 × 10^2^ copies/mL	Negative
EBV-DNA	<50.00 copies/mL	Negative
BK virus nucleic acid quantification	3.46 × 10^5^ copies/mL	1.14 × 10⁵ copies/mL
John cunningham viral load	Negative	
anti-dsDNA antibody	Negative	
anti-nuclear antibody	Negative	
T-cell count		
CD4 cell subset	24.95%	41.33%
CD8 cell subset	46.68%	44.97%
CD4/CD8 ratio	0.53	0.92
CD3 count	58 cell/*μ*L	932 cell/μL
CD4 count	20 cell/μL	430 cell/μL
CD8 count	37 cell/μL	468 cell/μL
Cerebrospinal fluid test		
Cerebrospinal fluid pressure	>320 mmH₂O	
Routine examination	Normal	
Biochemistry	Normal	
India ink staining	Negative	
Cryptococcal antigen test	Negative	
IgG	Normal	
Sterile body fluid culture	Negative	
Next-generation sequencing (Number of Sequence Reads)		
BK virus	3	
Streptococcus oralis	10	
Torque teno virus	99	
ADAMTS13 activity	95.9%	
ADAMTS13 inhibitor titer	0	
Soluble C5b-9 (sC5b-9)	Elevated	
Anti-complement factor H (anti-CFH) antibody	Positive	

**Figure 2:**
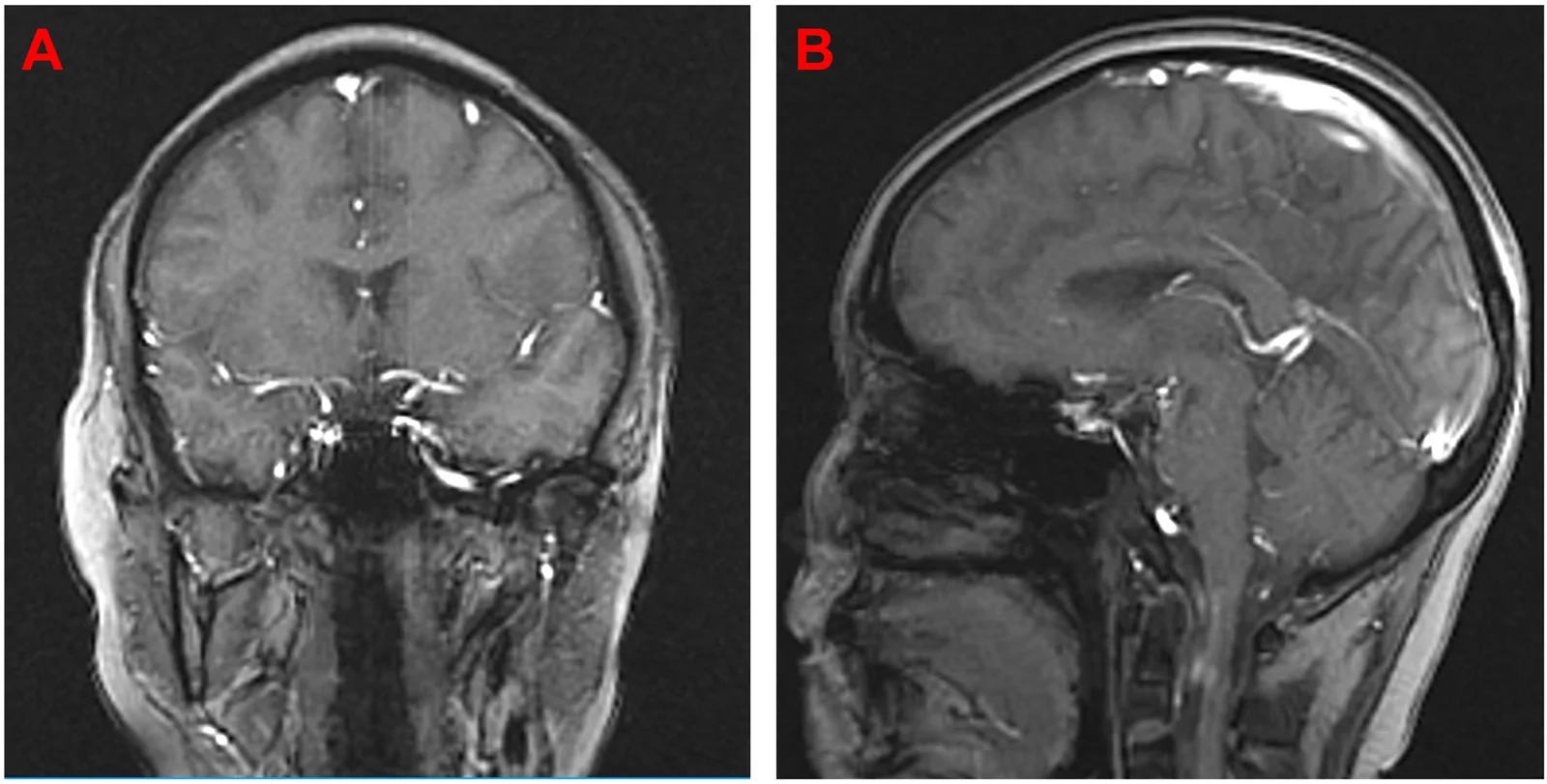
Contrast-enhanced brain MRI. **(A)** Coronal contrast-enhanced T1-weighted image; **(B)** Sagittal contrast-enhanced T1-weighted image.

**Figure 3:**
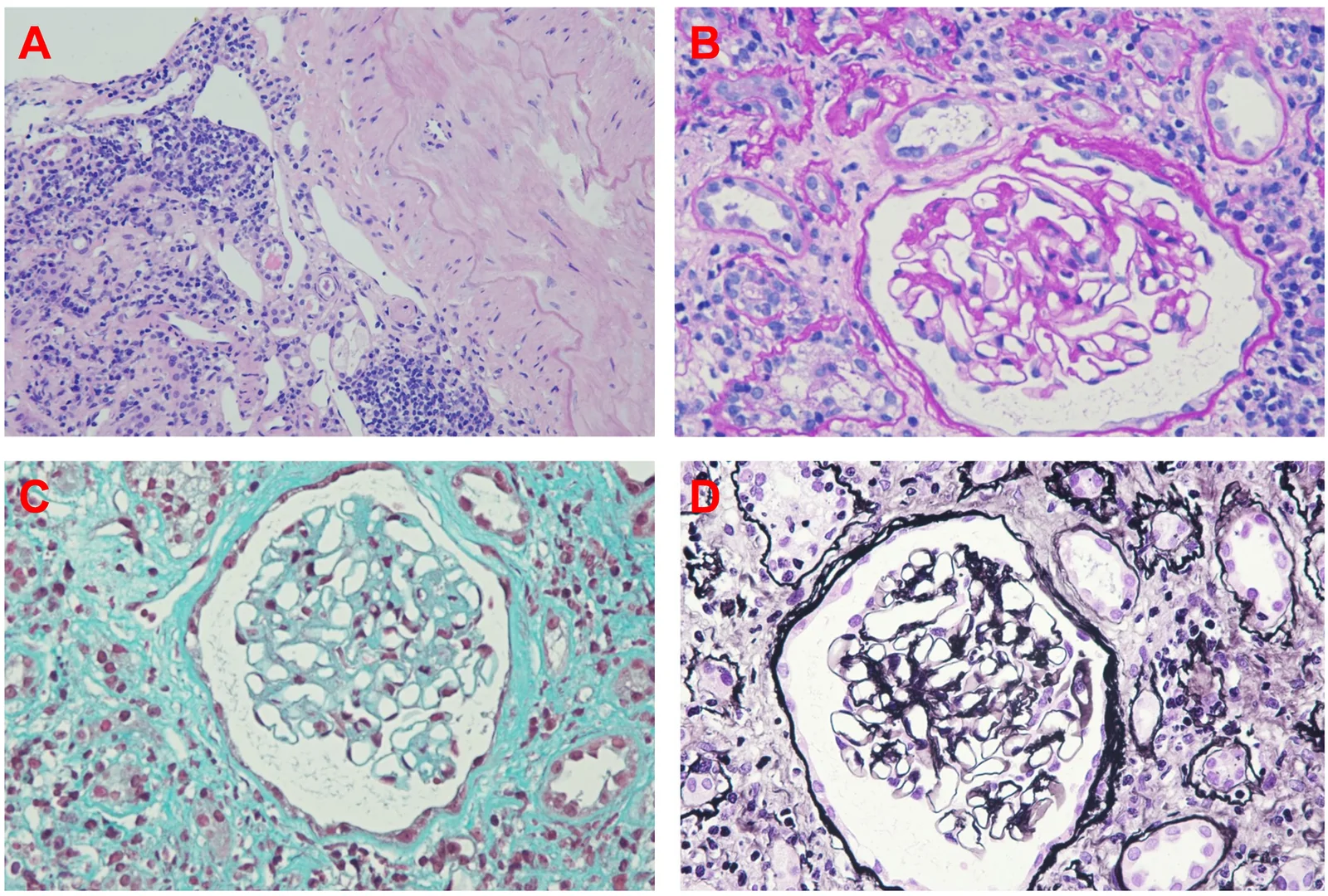
Histopathological findings of renal allograft biopsy. **(A)** HE staining (×200). A total of 24 glomeruli are identified, 16 of which show global sclerosis. Four glomeruli present fibrous thickening of Bowman's capsule. The mesangium shows segmental mild hypercellularity and matrix expansion (mm0). Two glomeruli exhibit capsular adhesion and segmental sclerosis. The basement membrane shows ischemic wrinkling. No glomerulitis is detected (g0). Approximately 80% of renal tubules are atrophic (ct3). Tubular epithelial cells show moderate to severe degeneration. Protein casts are seen in some tubular lumens. Tubulitis is present (t3). About 60% of the interstitium shows fibrosis (ci3) accompanied by infiltration of lymphocytes, monocytes and plasma cells (i3; i-IFTA3). The arteriolar and small arterial walls have moderate to severe thickening (ah0, CV3, V0). Peritubular capillaritis is noted (ptc2). **(B)** PAS staining ×400. The mesangium shows segmental mild hypercellularity and matrix expansion (mm0). Capsular adhesion and segmental sclerosis are present. The glomerular basement membrane exhibits ischemic wrinkling. The tubular basement membrane is thickened. **(C)** Masson's trichrome staining (×400). Fibrous thickening of Bowman's capsule is visible. About 60% interstitial fibrosis (ci3) is demonstrated. Approximately 80% tubular atrophy (ct3) is observed. The arteriolar and small arterial walls show moderate to severe thickening. **(D)** PASM staining (×400). The glomerular basement membrane shows ischemic wrinkling. Segmental sclerosis is present. The tubular basement membrane is thickened.

## Follow-up

BK viral load became undetectable 3 months after hospital discharge, and the anti-CFH antibody titer gradually decreased to below the lower limit of the reference range within 6 months after disease onset. Tacrolimus trough concentrations were maintained within the target range, and mizoribine was administered at a dose of 100 mg orally every morning and 50 mg orally every evening. At the 1-year follow-up, the patient developed progressive deterioration of allograft renal function and was initiated on dialysis. The patient has remained on dialysis for 2 months to date with good clinical status.

## Discussion

This case represents a rare and complex clinical scenario occurring 6 years after kidney transplantation, in which the patient presented with concurrent late-onset BKPyVAN complicated by viral encephalitis, AMR, and complement-mediated aHUS. The pathophysiological mechanisms of these three entities overlap, while their therapeutic principles are inherently conflicting. Following individualized treatment planning via multidisciplinary collaboration, the patient's condition was effectively controlled. This case fully illustrates the complexity of late allograft complications and carries important reference value for clinical practice.

BK polyomavirus infection is one of the most prevalent viral complications after kidney transplantation. Previous studies have demonstrated that the peak incidence of BKPyVAN occurs 1–2 years post-transplant, which is closely associated with cellular immunodeficiency induced by intensive early immunosuppression (14, 15). In recent years, there has been a growing number of reports of late-onset BKPyVAN, defined as disease onset beyond 5 years after transplantation. These late-presenting cases often occur in the context of immunosuppression regimen adjustments and persistent CD4⁺ T-cell lymphopenia, and are pathologically characterized by a higher propensity for chronic interstitial fibrosis and tubular atrophy, along with a significantly higher risk of graft loss (16). In our case, the disease developed 6 years after transplantation, accompanied by marked peripheral CD4⁺ T-cell depletion. Allograft biopsy revealed 60% interstitial fibrosis and 80% tubular atrophy, which is fully consistent with the clinicopathological features of late-onset BKPyVAN reported in the literature. Disseminated BK polyomavirus infection involving the central nervous system is extremely rare in kidney transplant recipients. In the existing literature, BK polyomavirus encephalitis is predominantly described in hematopoietic stem cell transplant recipients (17–19), with only scattered case reports in the kidney transplant setting (8, 9). BK virus encephalitis after kidney transplantation typically presents with seizure and altered consciousness as initial manifestations. The classic cerebrospinal fluid profile shows elevated protein with normal leukocyte count, and brain MRI may show no specific lesions or only mild meningeal thickening, which is fully concordant with the clinical findings in our patient (8). Rocha et al. previously reported a pediatric case of concurrent BKPyVAN and meningoencephalitis after kidney transplantation, highlighting that when transplant recipients present with renal function deterioration accompanied by neuropsychiatric symptoms, disseminated BK virus infection should be highly suspected, and cerebrospinal fluid BK viral load testing should be performed promptly (9).

The patient also presented with hemolytic anemia, thrombocytopenia, and progressive renal function deterioration, with schistocytes visible on peripheral blood smear, consistent with the clinical phenotype of TMA. Differential diagnoses included calcineurin inhibitor-induced thrombotic microangiopathy. However, this diagnosis was deemed less likely given the subtherapeutic tacrolimus trough level (10, 20). Renal allograft biopsy revealed no fibrin microthrombi within the glomerular or arteriolar lumina, endothelial cell necrosis, mucoid intimal thickening of small arteries, or characteristic lesions of acute CNI-associated TMA (21). Further complement workup revealed elevated C5b-9, positive anti-CFH antibodies, and normal ADAMTS13 activity, establishing the diagnosis of complement-mediated atypical hemolytic uremic syndrome. Basic research has confirmed that abnormally activated alternative complement pathway forms membrane attack complexes, which directly injure glomerular endothelial cells and induce microthrombosis. This constitutes the core pathogenic mechanism of complement-mediated aHUS (22). In addition, autoantibody-mediated dysfunction of complement regulatory proteins is also an important etiology of aHUS in adults (23).

Epidemiological data show that the overall incidence of *de novo* TMA after kidney transplantation is 3.20%, of which aHUS accounts for approximately 15%. Compared with other TMA subtypes, aHUS is associated with significantly inferior graft survival (24). Infection is a well-recognized trigger for aHUS onset. Both viral and bacterial infections can initiate the disease process by activating endothelial cells and inducing excessive activation of the alternative complement pathway (25–27). Given the simultaneous occurrence of BK viremia and aHUS in this case, we speculate that BK virus infection may act as an initiating factor, triggering aHUS through damaging renal tubular epithelial cells and activating endothelial cells and the complement cascade, and that the two conditions act synergistically to accelerate allograft function deterioration. Renal allograft ultrasonography plays a particularly critical role in the evaluation of renal allograft dysfunction in such cases of chronic allograft injury, as it enables initial screening of allograft structural abnormalities (28, 29).

The core therapeutic dilemma in this case lies in the fundamental conflict between the treatment principles of the three conditions. The cornerstone of treatment for BK virus infection is reduction of immunosuppression to restore antiviral cellular immunity (30). In contrast, AMR requires intensified immunosuppression to eliminate circulating antibodies, and aHUS requires complement inhibition or immunomodulatory therapy, both of which necessitate enhanced immune regulation (16, 23, 31). Although eculizumab is the standard first-line therapy for anti-CFH antibody-mediated aHUS, its use was substantially limited in this patient due to concurrent active BK virus encephalitis and BKPyVAN. First, as the terminal complement membrane attack complex serves as a core effector of innate immunity against polyomaviruses, complement blockade would significantly impair viral clearance and elevate the risk of infection dissemination and neurological deterioration (32). Second, severe chronic allograft injury had been confirmed by renal allograft biopsy, leaving very limited renoprotective benefit from complement inhibition. Third, the substantial financial burden of eculizumab was also an important consideration in clinical decision-making. For these reasons, the clinical team did not initiate complement inhibitor therapy and instead selected plasma exchange as the core acute-phase alternative. Through multidisciplinary consultation involving the departments of nephrology, neurology, infectious diseases, and hematology, a comprehensive treatment strategy was established, with plasma exchange as the core, immunosuppressant regimen adjustment as the foundation, and multisystem symptomatic support as the adjunct. Specifically, plasma exchange simultaneously targeted both AMR and aHUS, it removed circulating donor-specific antibodies to control antibody-mediated rejection, and eliminated anti-CFH autoantibodies, activated complement fragments, and inflammatory mediators to block complement-mediated endothelial injury (33). Switching mycophenolate mofetil to mizoribine preserves baseline immunosuppressive intensity and reduces rejection risk, while mitigating excessive suppression of cellular immunity to facilitate BK virus control (16). Concomitant symptomatic therapies including antiviral agents, intracranial pressure-lowering agents, and antiepileptic drugs were administered to manage central nervous system involvement. With this regimen, the patient achieved a favorable short-term prognosis, neuropsychiatric symptoms resolved completely by hospital day 33, with gradual stabilization of hematological parameters and renal function. However, in the absence of long-term maintenance complement inhibition, the patient remains at potential long-term risk of aHUS recurrence and progressive chronic allograft injury (33–35). Graft loss one year after initial presentation resulted from multiple factors. Severe chronic allograft injury at diagnosis, which comprises 60% interstitial fibrosis, 80% tubular atrophy and 16 globally sclerosed glomeruli out of 24, forms the fundamental pathological basis, as such extensive fibrosis is inherently progressive and irreversible. The concurrent BK virus infection, complement-mediated aHUS and subclinical antibody-mediated rejection exert synergistic tissue damage, as fibrogenic repair persists even after viral clearance, sustained alternative complement pathway activation drives progressive microvascular endothelial injury, and subclinical rejection further amplifies fibrogenesis. Meanwhile, the therapeutic conflict posed by active BK virus infection precludes adequate intensification of immunosuppression for rejection control and long-term complement blockade, leaving subchronic microvascular injury unaddressed and accelerating allograft functional decline. This case underscores the importance of regular long-term screening to enable early intervention before extensive irreversible chronic injury develops. The clinical course of this case fully validates the value of the multidisciplinary collaborative management model for complex allograft complications after kidney transplantation.

## Conclusion

The etiologies of progressive renal allograft dysfunction in the late post-transplant period are complex and multifactorial. In addition to well-recognized causes such as chronic rejection and calcineurin inhibitor nephrotoxicity, the potential for multiple overlapping pathologies including infection, rejection, and thrombotic microangiopathy should be actively considered. For rare cases of late-onset BK virus infection with central nervous system involvement concomitant with complement-mediated aHUS, prompt multidisciplinary consultation and the formulation of individualized treatment regimens that balance viral control and allograft preservation are critical to optimizing patient outcomes.

## Data Availability

The original contributions presented in the study are included in the article/Supplementary Material, further inquiries can be directed to the corresponding author/s.
